# Efficacy of tension-free vaginal tape compared with transobturator tape in the treatment of stress urinary incontinence in women: analysis of learning curve, perioperative changes of voiding function

**DOI:** 10.1186/1471-2490-11-13

**Published:** 2011-07-04

**Authors:** Hiroki Ito, Hiroyuki Yamanaka, Masayuki Hagiwara, Toru Furuuchi, Kazuhiro Matsumoto, Kunimitsu Kanai, Kiichiro Kodaira, Akiharu Ninomiya, So Nakamura

**Affiliations:** 1 Department of Urology, Yokohama City University Graduate School of Medicine and School of Medicine, Yokohama, Japan; 2 Department of Urology, Saiseikai Central Hospital, Tokyo, Japan

**Keywords:** Stress urinary incontinence, TVT surgery, TOT surgery

## Abstract

**Background:**

In this study, by comparing TVT surgery and TOT surgery for stress urinary incontinence in women, the characteristics and learning curves of both operative methods were studied.

**Methods:**

A total of 83 women with stress urinary incontinence treated with tension-free vaginal tape (TVT) (n = 38) or transobturator tape (TOT) (n = 45) at Saiseikai Central Hospital between April 2004 and September 2009 were included. We compare the outcomes and learning curves between TVT surgery and TOT surgery. In statistical analysis, Student's t test, Fisher's exact test, and Mann-Whitney's U test were used.

**Results:**

The surgical durations were 37.4 ± 15.7 minutes with TVT surgery and 31.0 ± 8.3 minutes with TOT surgery. A longer period of time was required for TVT surgery (p = 0.025). The residual urine at post-operative day 1 was higher in TVT surgery (25.9 ± 44.2 ml) than in TOT surgery (10.6 ± 19.2 ml) (p = 0.0452). The surgical duration of TVT surgery was shortened after the operator had performed 15 operations (p = 0.019).

**Conclusions:**

In comparison of TVT surgery and TOT surgery, the surgical duration of TVT surgery was longer and the residual urine of TVT surgery was higher at post-operative day 1. Surgical experience could shorten the duration of TVT surgery.

## Background

Midurethral slings have revolutionized the surgical management of stress urinary incontinence in women[1], and several procedures for midurethral slings have been reported. The highly safe and effective TVT surgery was established by Ulmsten[2] et al. in 1993 and TOT surgery was subsequently developed by Delorme[3] et al. in 2001. Comparisons between TVT surgery and TOT surgery have been reported mainly in Europe and the U.S.A., but their relative reported merits regarding safety and efficacy vary depending on the researchers[4-8].

At our hospital, by comparing TVT surgery and TOT surgery for stress urinary incontinence in women, the characteristics and learning curves of both operative methods were studied.

## Methods

The subjects consisted of 38 cases of TVT surgery and 45 cases of TOT surgery performed at Saiseikai Central Hospital during the period between April 2004 and September 2009. This trial was conducted in accordance with the principles espoused by the Declaration of Helsinki and all local regulations. This study was approved by the ethical review board of Saiseikai Central Hospital. All patients provided written informed consent. We used a 1-hour pad test, a urine flow measurement test, and residual urine determination for all patients in this study. In addition, we asked the patients to answer a questionnaire about incontinence symptoms and QOL (ICI Questionnaire-Short Form; ICIQ-SF) (Table 1).

**Table 1:** Patient pre-operative general characteristics; mean ± SD and n(%)

	TVT surgery	TOT surgery	p Value
Number	38	45	
Age	59.8 ± 10.0	62.6 ± 11.2	0.237*
BMI	23.1 ± 2.4	24.6 ± 4.1	0.0477*
Parity	2.0 ± 0.8	2.0 ± 0.9	0.9241*
Menopause	28(73.7%)	34(75.6%)	> .9999**
POP	7(18.4%)	15(33.3%)	0.2048**
UUI	9(23.7%)	10(22.2%)	> .9999**
post-hysterectomy	5(13.2%)	7(15.6%)	> .9999**
Pad test (g)	38.5 ± 36.6	37.8 ± 44.4	0.9479*
maximum urine flow rate (ml/s)	32.1 ± 13.4	30.7 ± 11.9	0.6509*
average urine flow rate (ml/s)	24.1 ± 11.0	22.4 ± 10.5	0.5044*
voided volume (ml)	299.5 ± 90.8	290.3 ± 85.8	0.6543*
post-voiding residue (ml)	15.9 ± 31.9	13.8 ± 25.3	0.7472*
ICIQ-SF	15.3 ± 4.2	13.6 ± 4.8	0.1162*
observation period (day)	121.6 ± 144	93.8 ± 94.9	0.3165*

* Student's t test ** Fisher's exact test

The operative method involved TVT surgery performed during the period between April 2004 and December 2006; after the introduction of TOT surgery to our hospital in December 2006, TOT surgery was generally performed in cases. Recently, many reports on the efficacy of TVT surgery for severe incontinence[8-10] have been published, so TVT surgery was performed in a total of 5 cases of incontinence with severe symptoms in May 2009 and thereafter. There were a total of 6 cases with POP (total vaginal hysterectomy in 1 case, surgery to form an anterior wall in 2 cases, TVM (tension-free vaginal mesh) surgery in 3 cases), in which a single-stage radical operation was performed at the time of urethral sling surgery. The surgery for the study was performed by the same surgeon, and for the anesthesia technique, local anesthesia and intravenous anesthesia were concomitantly used. A cough test was conducted as a stress test during surgery, and urethral depression during TVT surgery was not performed. During TOT surgery, the puncture site was at the clitoral level of the inferior ramus of the pubic bone and the sling of a Gynecare TVT device was used. An outside-in method was used with an Emmet needle (LANDANGER, France) in all cases.

Both surgeries required hospitalization of 3 days and 2 nights, with admission on the day prior to surgery and discharge on the day following surgery. The catheter was removed on the day following surgery in all cases unless urinary retention appeared. The patients were permitted to be discharged after their urination condition was checked. Even though the patient could be discharged from a medical perspective, if he/she was too elderly or was strongly anxious to stay at the hospital, hospitalization was extended by 1 to 2 days.

As items for comparison between the two operative methods, the following were studied: surgical duration, number of days of catheter placement, number of days of hospitalization, results of urine flow measurement on post-operative day 1 and in post-operative month 3, ICIQ-SF (incontinence symptoms and QOL questionnaire) value in post-operative month 3, and perioperative and postoperative complications.

For comparing the 2 groups of TVT surgery and TOT surgery, Student's *t-*test was used for the perioperative and postoperative complications and Fisher's exact test was used for the other items.

Learning curves were presumed on the basis of transitions of the surgical duration of TVT surgery and TOT surgery and Mann-Whitney's U test and Fisher's exact test were used for the items of surgical duration, hemorrhage volume, number of days of catheter placement, number of days of hospitalization, post-operative urinary residual volume, and post-operative complications, in order to study the adequacy.

## Results

Surgical performance was compared (Table 2). In the comparison of surgical duration, the value was 37.4 ± 15.7 minutes with TVT surgery and 31.0 ± 8.3 minutes with TOT surgery. A longer period of time was required for TVT surgery (p = 0.025). The hemorrhage volume was 0.9 ± 5.1 ml with TVT surgery and 3.1 ± 10.2 ml with TOT surgery (p = 0.2427). The duration of catheter placement and the number of days of hospitalization were 2.4 ± 1.4 days and 3.3 ± 0.9 days with TVT surgery and 2.0 ± 0.2 days (p = 0.1020) and 3.3 ± 0.7 days (p = 0.7748) with TOT surgery, respectively.

**Table 2:** Clinical results of both TVT surgery and TOT surgery

		TVT surgery	TOT surgery	p Value
surgical duration (min)		37.4 ± 15.7	31.0 ± 8.3	0.0253*
blood loss (ml)		0.9 ± 5.1	3.1 ± 10.2	0.2427*
catheterization (day)		2.4 ± 1.4	2.0 ± 0.2	0.1020*
admission (day)		3.3 ± 0.9	3.3 ± 0.7	0.7748*
subcutaneous hematoma		2(5.3%)	1(2.2%)	0.5917**
post-operative urinary retention		3(7.9%)	0	0.1765**
	maximum urine flow rate (ml/s)	14.0 ± 6.8	13.4 ± 5.5	0.6477*
post-operative day 1	average urine flow rate (ml/s)	8.9 ± 5.5	8.6 ± 4.5	0.7798*
	post-voiding residue (ml)	25.9 ± 44.2	10.6 ± 17.2	0.0452*
	maximum urine flow rate (ml/s)	18.5 ± 7.7	16.5 ± 10.9	0.4245*
post-operative month 3	average urine flow rate (ml/s)	11.9 ± 6.6	9.1 ± 6.1	0.1059*
	post-voiding residue (ml)	33.4 ± 56.9	26.2 ± 48.4	0.577*
post-operative ICIQ-SF		1.5 ± 2.6	3.2 ± 4.8	0.1152*
post-operative dysuria		6(15.8%)	1(2.2%)	0.0574**
recurrence		0	1(2.2%)	> .9999**
erosion		0	1(2.2%)	> .9999**

* Student's t test ** Fisher's exact test

In the comparison of urine flow measurement, the urinary residual volumes on post-operative day 1 were 25.9 ± 44.2 ml with TVT surgery and 10.6 ± 19.2 ml with TOT surgery. The urinary residual volume on post-operative day 1 was higher for TVT surgery (p = 0.0452). However, the urinary residual volumes in post-operative month 3 were 33.4 ± 56.9 ml with TVT surgery and 26.7 ± 48.4 ml with TOT surgery, between which there was no significant difference (p = 0.577).

The peak urine flow rate and the mean urine flow rate on post-operative day 1 were 14.0 ± 6.8 days and 8.9 ± 5.5 days with TVT surgery and 13.4 ± 5.5 days (p = 0.6477) and 8.6 ± 4.5 days (p = 0.7798) with TOT surgery, respectively. The peak urine flow rate and the mean urine flow rate in post-operative month 3 were 18.5 ± 7.7 days and 11.9 ± 6.6 days with TVT surgery and 16.5 ± 10.9 days (p = 0.4245) and 9.1 ± 6.1 days (p = 0.1059) with TOT surgery, respectively.

The post-operative ICIQ-SF values were 1.5 ± 2.6 with TVT surgery and 3.2 ± 4.8 (p = 0.1152) with TOT surgery.

Regarding complications, with TVT surgery, post-operative subcutaneous hematoma was observed in 2 cases (2.5%) and post-operative urinary retention was observed in 3 cases (7.9%). On the other hand, with TOT surgery, postoperative subcutaneous hematoma was observed in 1 case (2.2%) and mucosal erosion was observed in 1 case (2.2%). In all cases, remission was obtained by conservative treatment such as follow-ups and extension of catheter placement. Dysuria was observed after surgery in 6 cases (15.8%) with TVT surgery and 1 case (2.2%) with TOT surgery (p = 0.0547). Bladder mispuncture, intestinal penetration, nerve and vascular damage, wound infection, sling exposure, and de novo urgency were not observed in either group. Postoperative recurrence was observed in 1 case with TOT surgery.

**Figure 1:**
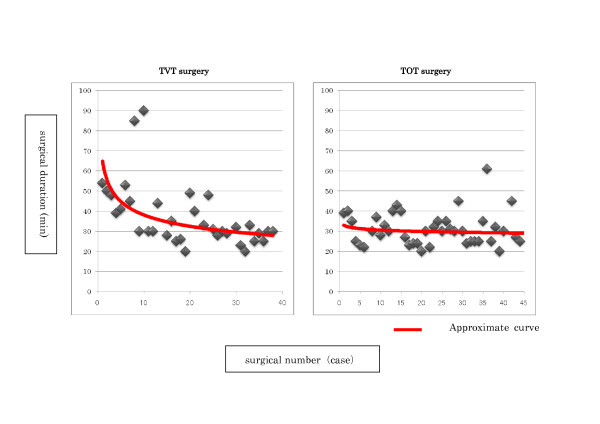
**Transitions of the surgical duration and the approximate curve of both TVT surgery and TOT surgery**.

Learning curves were created for the transitions of the surgical duration of TVT surgery and TOT surgery using Microsoft Office Excel 2007 (Figure 1). On the basis of the shape of the approximate curve, it was considered that proficiency would be obtained after around 15 cases in both groups. Therefore, each surgery group was divided into 3 phases: Phase I (1 to 15 cases), Phase II (16 to 30 cases), and Phase III (over 31 cases), and shortened surgical duration was consequently observed between Phase I (47.6 ± 19.1 minutes) and Phase II (32.8 ± 8.5 minutes) in TVT surgery (comparison between Phases I and II: p = 0.0186, comparison between Phases II and III: p = 0.0888). In TOT surgery, no shortened surgical duration was observed in either phase (comparison between Phases I and II: p = 0.1266, comparison between Phases II and III: p = 0.9816). The results of the comparison of the number of days of catheter placement, number of days of hospitalization, hemorrhage volume, urinary residual volume on post-operative day 1, and urinary residual volume in post-operative month 3 in each phase showed no obvious difference between TVT surgery and TOT surgery. Although the items of subcutaneous hematoma and post-operative urinary retention were studied, no difference was observed in any phase (Table 3).

**Table 3:** Analysis of learning curve: comparison between TVT surgery and TOT surgery

TVT surgery					
	**Phase I (1-15cases)**	**Phase II (16-30 cases)**	**Phase III (31-38cases)**	**p Value (comparison between I and II)**	**p Value (comparison between II and III)**

surgical duration (min)	47.6 ± 19.1	32.8 ± 8.5	26.9 ± 4.3	0.0186	0.0888
blood loss (ml)	2.1 ± 8.0	0	0	0.5124	0.7721
catheterization (day)	2.9 ± 2.1	2.1 ± 0.3	2.0 ± 0	0.5281	0.4915
admission (day)	3.4 ± 0.8	3.1 ± 0.3	3.6 ± 1.4	0.7524	> .9999
POD1 post-voiding residue (ml)	46.3 ± 62.1	15.2 ± 20.5	34.0 ± 7.5	0.424	0.823
POM3 post-voiding residue (ml)	41.7 ± 68.7	31.5 ± 49.0	3.3 ± 5.8	0.384	0.233
subcutaneous hematoma	1 (6.7%)	1 (6.7%)	0	0.9565*	0.4214*
post-operative urinary retention	2 (13.3%)	1 (6.7%)	0	0.5859*	0.4214*

**TOT surgery**					

	**Phase I (1-15 cases)**	**Phase II (16-30 cases)**	**Phase III (31-45 cases)**	**p Value (comparison between I and II)**	**p Value (comparison between II and III)**

surgical duration (min)	33.2 ± 7.0	29.2 ± 6.4	30.7 ± 11.1	0.1266	0.9816
blood loss (ml)	1.4 ± 5.4	2.0 ± 7.8	6.2 ± 15.6	> .9999	0.7297
catheterization (day)	2.0 ± 0	2.0 ± 0	2.1 ± 0.3	0.5705	0.8538
admission (day)	3.1 ± 0.5	3.3 ± 0.8	3.3 ± 0.9	> .9999	0.6784
POD1 post-voiding residue (ml)	4.5 ± 15.0	12.9 ± 17.3	20.3 ± 26.8	0.135	0.423
POM3 post-voiding residue (ml)	10.4 ± 20.8	38.9 ± 67.0	29.0 ± 41.2	0.165	0.951
subcutaneous hematoma	0	0	1 (6.7%)	> .9999*	0.274*
post-operative urinary retention	0	0	0	> .9999*	> .9999*

p value: Mann-Whitney's U test * Fisher's exact test

## Discussion

TVT surgery involved the procedures of penetrating the paraurethral and retropubic cavity with a needle. These penetrations were blind procedures. The operators had no obvious target in terms of where to penetrate with the needle. Therefore, these were difficult techniques for inexperienced operators. Although TOT surgery also involved blind penetrating procedures, there was an obvious target, namely, the obturator foramen. This might make TOT surgery easier than TVT surgery for inexperienced operators. With TVT surgery, cystoscopy is essential after puncture. On the other hand, with TOT surgery, which is less likely to involve bladder damage, it is not essential. These differences could explain the difference in surgical duration between the two groups.

In the comparison of the urinary residual volume on post-operative day 1, it was higher with TVT surgery than TOT surgery. This does not conflict with previous reports by researchers[7-9], and it is commonly understood that, because the angle of the curve of the suburethral hammock is more acute with TVT surgery, a higher urinary residual volume is observed. However, a dissection around the urethra is performed more widely with TOT surgery. Therefore, it is natural that edematous changes in the urethra after surgery would become predominant with TOT surgery, which could contribute to increased residual urine. The results of the present study indicate that the range of dissection did not influence the residual urine.

No differences were observed between both surgeries in comparison of the urinary residual volume in post-operative month 3. Chene[11] et al. measured the angles of slings placed in TVT surgery and TOT surgery several months after the surgeries. The values were 116.3 ± 6.62° with TVT surgery (n = 28) and 130.75 ± 7.23° with TOT surgery (n = 30). A significantly more acute angle was observed with TVT surgery (p = 0.001). Despite the fact that the angle of the curve of the suburethral hammock was unchanged, there was no difference in the long-term urinary residual volume and, therefore, another factor other than the angle of the curve of the hammock must influence the long-term urinary residual volume. Midurethral sling surgery is performed to fix the urethra and involves few anatomic changes in surrounding tissues such as the bladder and external sphincter urethral muscle. The voiding functions and the cooperation system of the bladder and external sphincter urethral muscle would be adapted to post-operative urethral changes. This adaptation might reduce the difference between the two operative methods. In fact, Richter et al. [12] reported that there would be no large difference between TVT surgery and TOT surgery in terms of long-term results.

With TVT surgery, a learning curve in terms of the surgical duration was found to be around 15 cases. On the other hand, with TOT surgery, the presence of a learning curve was not confirmed in any of the cases studied. This might be because the surgeon had already completed the learning curve for TVT surgery (introduced in 2004 at our hospital) by the time TOT surgery was introduced at our hospital (2006). This study had a certain limitation. There was a bias because it would be more reasonable to test the learning curve of TOT surgery in urologists who do not have prior experience of TVT surgery. However, the results indicated that the technique of TVT surgery could be applied to TOT surgery. Moreover, a study on the items of number of days of catheter placement, number of days of hospitalization, hemorrhage volume, urinary residual volume on post-operative day 1, urinary residual volume in post-operative month 3, post-operative subcutaneous hematoma, and post-operative urinary retention except for surgical duration revealed that both operative methods yield excellent outcomes in the early stages. The absence of a learning curve also indicated that both operative methods could be performed safely in the early stages of the introduction of surgery and could consistently obtain excellent surgical performance.

In this study, there was no difference in the post-operative ICIQ-SF value and the response rate of the two surgeries. However, the post-operative ICIQ-SF values were 1.48 ± 2.63 with TVT surgery and 3.16 ± 4.79 with TOT surgery, showing viability. This might be because the cases of UUI as a complication accounted for 20% or more in each group and, therefore, the ICIQ-SF value, which indicates the QOL impairment of overall incontinence numerically, showed a wide range even after surgery.

For TVT surgery, there is a case report that patients died due to intestinal puncture. In the present study, no serious complications such as bladder mispuncture, nerve and vascular damage, wound infection, or exposure of the sling occurred in either group. Moreover, de novo urgency, which has been reported as highly frequent, at 0.8 to 47.2%[13,14], was not observed in this study. In terms of surgery for not malignancy but rather QOL disorder, these midurethral sling surgeries should naturally be required to be highly safe. The present study indicated that short- and long-term safety levels were high.

As is clear from the results of the present study, the efficacy and safety of TVT surgery and TOT surgery are generally well established, and few differences were observed in the frequency of complications between the selected operative methods. There will always be a debate over which operative method is best to be selected for those with little experience of midurethral sling surgery in the future. However, we have demonstrated that the introduction procedure (starting with TVT surgery followed by introduction of TOT surgery) at our institution can obtain satisfactory surgical performance.

Post-operative recurrence was observed in only 1 case of TOT surgery. An improvement of stress urinary incontinence was observed immediately after surgery, but a recurrence of stress urinary incontinence was observed after post-operative month 3. There is a report in which aging is defined as a risk factor of post-operative recurrence of stress urinary incontinence; however, the case of recurrence that we encountered this time was a case of a 62-year-old female who had no particular previous history. There are few reports on the predictive factors of post-operative recurrence and poor surgical effect[15], and it will be necessary to clarify them in future studies. Moreover, for unsuccessful cases of TOT surgery, there is a report that an improvement of urinary incontinence was observed by performing TVT surgery subsequently[16]. The development of additional treatment for unsuccessful cases is a goal for future study.

There are several limitations in this study. The limitations included a small number of the cases involved, a short post-operative follow-up duration, and poor examinations of pre- and post-operative voiding functions. We must analyze midurethral sling surgery more carefully on the basis of these results.

## Conclusions

In comparison of TVT surgery and TOT surgery, the surgical duration of TVT surgery was longer and the post-operative residual urine of TVT surgery was higher at post-operative day 1. With TVT surgery, a learning curve in terms of the surgical duration was found to last around 15 cases. Both operative methods could be performed safely in the early stages after the introduction of surgery and could consistently obtain excellent surgical performance. This study indicated that the technique of TVT surgery can also be applied to TOT surgery.

## Abbreviations

TVT: tension-free vaginal tape; TOT: transobturator tape; POP: pelvic organ prolapsed; ICIQ-SF: incontinence symptoms and QOL questionnaire.

## Competing interests

The authors declare that they have no competing interests.

## Authors' contributions

All authors participated in the design and conduct of the study. All authors reviewed and approved the final version of the manuscript.

## Pre-publication history

The pre-publication history for this paper can be accessed here:

http://www.biomedcentral.com/1471-2490/11/13/prepub
